# Effects of capsicum oleoresin supplementation on rumen fermentation and microbial abundance under different temperature and dietary conditions *in vitro*

**DOI:** 10.3389/fmicb.2022.1005818

**Published:** 2022-09-26

**Authors:** Zhigao An, Gan Luo, Mohamed Abdelrahman, Umair Riaz, Shanshan Gao, Zhiqiu Yao, Tingzhu Ye, Haimiao Lv, Jvnwei Zhao, Changzhi Chen, Liguo Yang

**Affiliations:** ^1^Key Laboratory of Animal Genetics, Breeding and Reproduction, Ministry of Education, College of Animal Science and Technology, Huazhong Agricultural University, Wuhan, China; ^2^International Joint Research Centre for Animal Genetics, Breeding and Reproduction (IJRCAGBR), Huazhong Agricultural University, Wuhan, China; ^3^Animal Production Department, Faculty of Agriculture, Assiut University, Asyut, Egypt; ^4^Faculty of Veterinary and Animal Sciences, Islamia University of Bahawalpur, Bahawalpur, Pakistan; ^5^Bright Farming Co. Ltd., Shanghai, China; ^6^Hubei Province’s Engineering Research Center in Buffalo Breeding and Products, Wuhan, China

**Keywords:** capsicum oleoresin, hyperthermal stress, dietary conditions, rumen fermentation, microbial abundance

## Abstract

This study aimed to determine the effect of capsicum oleoresin (CAP) on rumen fermentation and microbial abundance under different temperature and dietary conditions *in vitro*. The experimental design was arranged in a 2 × 2 × 3 factorial format together with two temperatures (normal: 39°C; hyperthermal: 42°C), two forage/concentrate ratios (30:70; 70:30), and two CAP concentrations in the incubation fluid at 20 and 200 mg/L with a control group. Regarding the fermentation characteristics, high temperature reduced short-chain fatty acids (SCFA) production except for molar percentages of butyrate while increasing acetate-to-propionate ratio and ammonia concentration. The diets increased total SCFA, propionate, and ammonia concentrations while decreasing acetate percentage and acetate-to-propionate ratio. CAP reduced acetate percentage and acetate-to-propionate ratio. Under hyperthermal condition, CAP could reduce acetate percentage and increase acetate-to-propionate ratio, lessening the negative effect of high heat on SCFA. Hyperthermal condition and diet altered the relative abundance of microbial abundance in cellulose-degrading bacteria. CAP showed little effect on the microbial abundance which only increased *Butyrivibrio fibrisolvens*. Thus, CAP could improve rumen fermentation under different conditions, with plasticity in response to the ramp of different temperature and dietary conditions, although hardly affecting rumen microbial abundance.

## Introduction

In the ruminants, the rumen functioning is susceptible to climatic and dietary changes. It was reported that temperature and feed characteristics (forage: concentration) are significant parameters that affect rumen function (Sales et al., 2021). Ruminal temperature is influenced by severe changes in climatic conditions (Lees et al., 2019; Sullivan et al., 2022). Fluctuations in ruminal temperature significantly affect rumen microbial abundance and short-chain fatty acids (SCFA) production (Uyeno et al., 2010; Wang et al., 2022). Similarly, diet is the other main factor affecting rumen fermentation variables which serves as the medium for the survival of rumen microorganisms (Mavrommatis et al., 2021a). The rumen is stressed more by using a high-concentrate diet, which is the common nutritional strategy under heat stress (Nasrollahi et al., 2019). Ruminal metabolic diseases caused by a high-concentrate diet jeopardizes productivity of animal (Tian et al., 2015). Therefore, new solutions are needed to be investigated in nutritional measures to maintain normal rumen physiology under high-temperature stress and high-concentrate ratios to improve ruminant productivity.

Capsicum oleoresin (CAP) is extracted from capsicum and the active ingredient is capsaicin. As a dietary additive, CAP has a variety of functions, such as anti-inflammatory (Ghiasi et al., 2019), antioxidant (Karadas et al., 2014), regulation of rumen fermentation (Temmar et al., 2021), and alteration of gut microbiota (Grazziotin et al., 2020) which may be beneficial to dairy cows. Meanwhile, CAP has been shown to be a functional additive in the regulation of metabolic diseases caused by high-concentrate diet and resist heat stress. It has been reported that CAP could affect rumen fermentation (Fandiño et al., 2008), alter feeding behavior, and may affect ruminal acidosis (Castillo-Lopez et al., 2021) in high-concentrate diet. Additionally, CAP against overheating of the body by thermo regulating (Szolcsányi, 2015) and probably affects productivity caused by heat stress. It has been shown CAP could reduce rectal temperature and improve productive performance in dairy cows (Da Silva, 2017) and pigs (Kroscher et al., 2022). Nevertheless, the CAP effect on rumen fermentation and microbial abundance is unclear at high-temperature and different dietary compositions, which limits the further application of CAP. In response to this situation, this study investigated the CAP effect on ruminal fermentation characteristics and microbial abundance under high heat conditions and different fermentation substrates.

## Materials and methods

### Experimental design and treatments

The experiment conditions were arranged in a 2 × 2 × 3 factor design, including two temperatures (normal: 39°C; hyperthermal: 42°C), two diet ratios [forage/concentrate ratio, 30:70 (low forage, LF); 70:30 (high forage, HF)], and two CAP concentrations in the incubation fluid at 20 (LC) and 200 mg/L (HC) with a control group (CON). Physiological temperature and high temperature were selected based on physiological rumen temperature (Eihvalde et al., 2016) and the maximum rumen temperature under heat stress (Beatty et al., 2008). Fermentation substrates were composed of whole corn silage and commercial concentrate. Corn silage and concentrate were dried at 65°C for 48 h and passed through a 1 mm sieve. The commercial CAP products are provided by Tianxu Food Additive Co. Ltd. (MY1098, 10.0% capsaicin, Guangzhou, China). The fermentation substrates are presented in Table 1.

**Table 1:** Chemical composition of the experimental diets.

Chemical composition	F:C = 30:70 (LF)^1^	F:C = 70:30 (HF)
Dry matter (%)	90.94	91.67
% of dry matter		
Organic matter	92.84	92.12
Crude protein	15.53	12.67
Neutral detergent fiber	30.34	45.19
Acid detergent fiber	18.77	25.27
Ash	7.16	7.88

1 C, forage/concentrate ratio.

### *In vitro* fermentations and sampling

Rumen fluid was collected from four cows immediately after slaughtering at a commercial slaughterhouse (Heng Xing Slaughterhouse, Wuhan, China). All cows were fed a total mixed diet mainly consisting of silage, excluding disease factors, and rumen fluid was collected individually through four layers of cheesecloth within 15 min after slaughter and reached the laboratory within 30 min. Rumen fluid was maintained in a preheated bottle without top air at 39°C after collecting until it reached the laboratory. Next, fresh buffer solutions were mixed according to Beatty et al. (2008)). Briefly, rumen fluid was mixed in equal proportion and added to the buffer at the ratio of 1:2 with 39°C under continuous CO_2_ flushing. Fermentation substrate (0.5 g) and incubation fluid (50 mL) were added to each fermentation flask, injected with CO_2_, and sealed with a butyl rubber plug and aluminum cap. Finally, the flask was placed in two constant temperature shock incubators set at 39°C and 42°C, respectively.

At the end of fermentation, the pH of the incubation fluid was measured with a pH meter (FE28 – Standard, Mettler Toledo, Switzerland). The collected culture medium was divided into 2 mL sterile tubes. Isometric samples were collected and stored at −20°C immediately for ammonium and SCFA analysis. The remaining samples were stored at −80°C with one isometric sample for further microbial abundance analysis and the remaining samples were retained.

### Chemical analysis

For SCFA analysis, incubation fluid (1 mL) was added to 200 μL of 25% metaphosphoric acid and centrifuged at 12,000 *g* for 10 min at 4°C to obtain the supernatant. Using a gas chromatograph (7890A, Agilent Technologies, Santa Clara, CA, United States), capillary column (30 m × 0.25 mm × 00.25 μm, DB – FFAP, Agilent Technologies, Santa Clara, CA, United States), and a pyrophoric detector. The oven temperature was initially maintained at 80°C for 5 min, increased to 190°C, 12.5°C/min, and held at 190°C for 3 min. The injector and detector temperatures were 250°C and 280°C, respectively. Determination of ammonia nitrogen was performed by colorimetry according to Weatherburn (1967).

The CH_4_ yield was calculated according to previously reported formula (Moss et al., 2000), considering a hydrogen recovery of 90% (default):

CH_4_ (mmol/L) = 0.45 × Acetate (mmol/L) – 0.275 × Propionate (mmol/L) + 0.40 × Butyrate (mmol/L).

### Microbial analysis

Microbial DNA was extracted from rumen fluid using a Tiangen commercial kit according to the instructions (DP328-02, Tiangen Biotechnology Co., Ltd, Beijing, China) as reported in a previous study (Ding et al., 2014), and DNA concentration and purity were determined by spectrophotometer. Microorganisms in the samples were determined by SYBR Green quantitative real-time polymerase chain reaction (qPCR) assay for different microbiota as described by Zhu et al. (2017)). Briefly, 5 μL of SYBR Green q PCR Master Taq (2×), 0.2 μL of each primer, 0.5 μL of genomic DNA (10 ng/μL), and double-distilled water were added to make a total volume of 10 μL. Specific primers were presented in Table 2. All primers amplification started with a denaturalization at 95°C for 3 min, followed by 40 cycles of 95°C for 5 s, 60°C for 30 s, and 72°C for 30 s. All qPCR assays were performed in triplicate for each sample.

**Table 2:** PCR primers for real-time PCR assay.

Target species	Primer sequence (5′)	GeneBank accession number	Size (bp)
Total bacteria	F: CGGCAACGAGCGCAACCC	AY548787.1	147
	R: CCATTGTAGCACGTGTGTAGCC		
Total anaerobic fungi	F: GAGGAAGTAAAAGTCGTAACAAGGTTTC	GQ355327.1	120
	R: CAAATTCACAAAGGGTAGGATGATT		
Total protozoa	F: GCTTTCGWTGGTAGTGTATT	HM212038.1	234
	R: CTTGCCCTCYAATCGTWCT		
Total methanogens	F: TTCGGTGGATCDCARAGRGC	GQ339873.1	160
	R: GBARGTCGWAWCCGTAGAATCC		
*Ruminococcus albus*	F: CCCTAAAAGCAGTCTTAGTTCG	CP002403.1	176
	R: CCTCCTTGCGGTTAGAACA		
*Ruminococcus flavefaciens*	F: ATTGTCCCAGTTCAGATTGC	AB849343.1	173
	R: GGCGTCCTCATTGCTGTTAG		
*Butyrivibrio fibrisolvens*	F: ACCGCATAAGCGCACGGA	HQ404372.1	65
	R: CGGGTCCATCTTGTACCGATAAAT		
*Fibrobacter succinogenes*	F: GTTCGGAATTACTGGGCGTAAA	AB275512.1	121
	R: CGCCTGCCCCTGAACTATC		
*Prevotella ruminicola*	F: CTGGGGAGCTGCCTGAATG	MH708240.1	102
	R: GCATCTGAATGCGACTGGTTG		
*Ruminobacter amylophilus*	F: GAAAGTCGGATTAATGCTCTATGTTG	LT975683.1	74
	R: CATCCTATAGCGGTAAACCTTTGG		

The relative population sizes of total bacteria, total anaerobic fungi, total protozoa, total methanogens, *Ruminococcus albus*, *Ruminococcus flavefaciens*, *Butyrivibrio fibrisolvens*, *Fibrobacter succinogenes*, *Prevotella ruminicola*, and *Ruminobacter amylophilus* were expressed as the proportion of 16S rDNA of total rumen bacteria at 24 h. Relative abundance was expressed as a proportion of the specific gene fragments number to the total rumen bacterial 16S rDNA number, calculated according to the following equation: relative quantification = 2^− (Ct target − Ct total bacteria)^, where C_t_ represents threshold cycle based on the report of Castagnino et al. (2015)).

### Statistical analysis

All statistical analyses were performed using the PROC MIXED procedure of SAS 9.4 (SAS Institute Inc.). Data were analyzed according to factorial permutations in a randomized compartmentalized design. The main effects of amylases include temperature, diet, and CAP levels, their 2-way, 3-way interactions, and the random effects. For daily measurements, the data were analyzed as repeated measurements. The composite symmetry was a variance–covariance structure. All values were presented as least squares mean ± standard error of the mean (SEM), stated otherwise. Comparisons between LS means were performed according to the PDIFF function. All experiments and analyses were made in triplicate. The analysis of variance followed by a Duncan test (*p* < 0.05) was used for the mean comparison and the trend was set at 0.05 < *p* ≤ 0.10.

## Results

### Rumen fermentation

The fermentation characteristics influenced by culture conditions and CAP addition are indicated in Table 3. SCFA decreased by hyperthermal condition and increased by high-concentrate diets (*p* < 0.05), while there was no effect on CAP addition. Hyperthermal condition increased the molar percentages of butyrate, isobutyrate, and acetate-to-propionate ratio while decreasing propionate and caproate percentage (*p* < 0.05). The addition of CAP decreased acetate percentage and acetate-to-propionate ratio (*p* < 0.05) and tended to increase propionate percentage (*p* = 0.09). Hyperthermal condition and high concentration diet increased ammonia concentrations (*p* < 0.05), and there was an interaction between temperature and diet (*p* < 0.05). Temperature tended to increase pH in the hyperthermal group compared to the normal group (*p* = 0.09). Temperature, diet, and CAP addition did not affect methane.

**Table 3:** Fermentation characteristics as affected by temperature, diet, Capsicum oleoresin (CAP) supplementation, and their interactions.

Item	Temperature (T)			Diet (D)^1^			Capsicum oleoresin (C)^2^			SEM	*p*-Value			Interaction^3^
	39°C	42°C		HF	LF		CON	LC	HC		T	D	C	
pH	6.66	6.72		6.68	6.70		6.66	6.71	6.71	0.02	0.09	0.65	0.43	(D × T × C)
Ammonia (mg/dL)	8.67	16.02		11.72	12.96		12.71	12.32	12.00	0.65	<0.001	0.01	0.27	D × C, (D × T × C)
CH_4_ (mmol/L)	11.15	11.80		11.24	11.71		11.71	11.84	10.87	0.32	0.30	0.44	0.41	
SCFA (mmol/L)	54.99	42.64		44.74	58.89		46.81	51.30	48.34	1.95	<0.001	0.02	0.49	(D × T)
SCFA proportion (mol/100 mol)														
Acetate	59.50	60.68		62.54	57.64		63.16^a^	59.25^b^	57.87^b^	0.81	0.38	0.001	0.01	(D × T)
Propionate	32.73	19.69		24.08	28.34		23.80	25.50	29.32	1.53	<0.001	0.04	0.09	
Butyrate	5.44	16.73		10.70	11.47		10.57	12.40	10.29	1.09	<0.001	0.51	0.28	
Isobutyrate	0.19	0.57		0.41	0.36		0.38	0.44	0.32	0.04	<0.001	0.34	0.17	D × T
Valerate	0.56	1.42		1.08	0.91		1.02	1.11	0.84	0.09	<0.001	0.08	0.09	D × T
Caproate	1.57	0.90		1.19	1.29		1.06	1.29	1.36	0.10	<0.001	0.48	0.20	D × T
Ratio of acetate to propionate	1.83	3.69		3.08	2.44		3.27^a^	2.86^ab^	2.15^b^	0.24	<0.001	0.05	0.02	T × C

1 Dietary conditions (forage/concentrate ratio): F:C, 30:70 (LF); F:C, 70:30 (HF).

2 Target CAP concentration (mg/L): CON, 0; LC, 20; HC, 200.

3 Listing only interactions are showing a significant effect (*p* ≤ 0.05) and, as shown in a bracket, a tendency (*p* ≤ 0.10).

a,b Means in the same row with different lowercase superscript letters are significantly different (*p* < 0.05).

**Figure 1:**
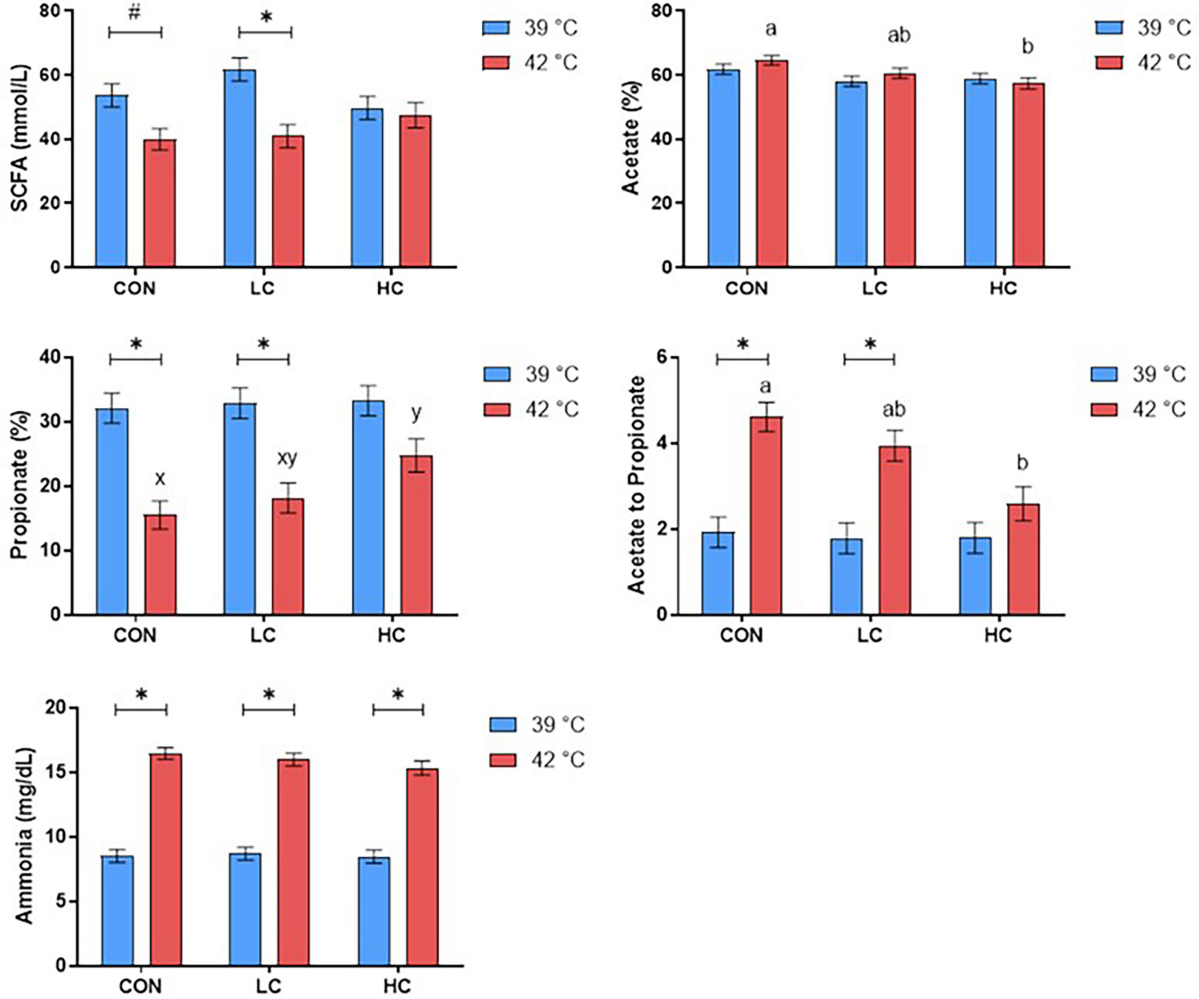
Concentrations of short-chain fatty acids (SCFA) and ammonia were affected by capsicum oleoresin (CAP) supplementation and incubation temperature. Least-square means of CAP groups within each temperature sharing no common letter differ at *p* ≤ 0.05 (a, b) or 0.05 < *p* ≤ 0.10 (x, y). Differences between the diet within each CAP group (*p* ≤ 0.05) (*) or 0.05 < *p* ≤ 0.10 (#). CAP levels: CON: 0 mg/L; LC: 20 mg/L; and HC: 200 mg/L.

Under hyperthermal condition, the CAP group reduced the SCFA in LC (*p* < 0.05) compared with the CON and HC (Figure 1). The HC group decreased acetate percentage and acetate to propionate ratio relative to the CON (*p* < 0.05) while tending to increase propionate percentage (*p* ≤ 0.10). At the same time, hyperthermal condition reduced propionate percentage and increased acetate to propionate ratio in the CON and LC compared with physiological temperature (*p* < 0.05), while there was no difference in the HC group.

**Figure 2:**
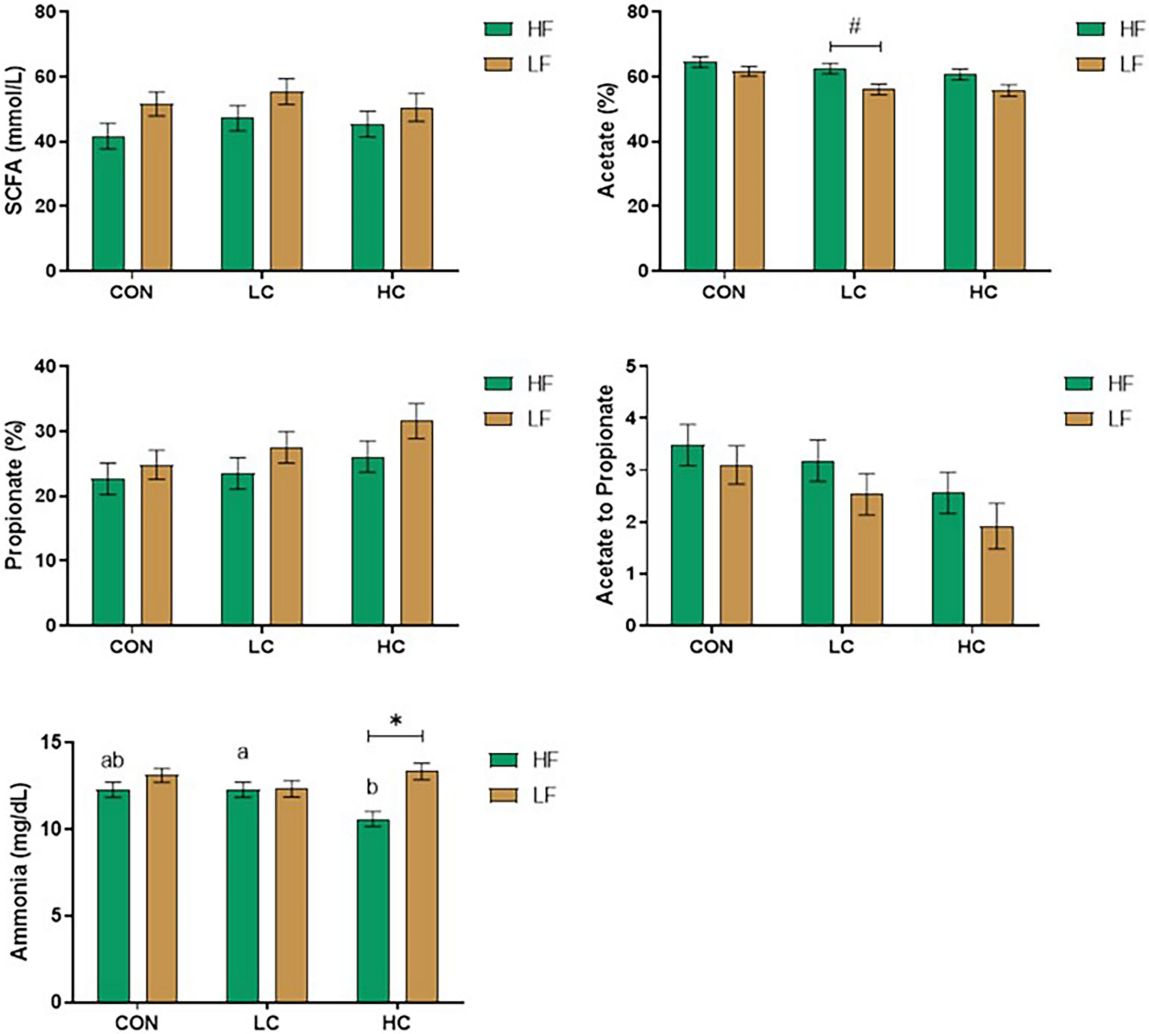
Concentrations of short-chain fatty acids (SCFA) and ammonia were affected by capsicum oleoresin (CAP) supplementation and diet. Least-square means of CAP groups within each diet sharing no common letter differ at *P* ≤ Concentrations. Differences between the dietary conditions within each CAP group (*p* ≤ 0.05) (*) or 0.05 < *p* ≤ 0.10 (#). Dietary conditions (forage/concentrate ratio): LF: 30:70; HF: 70:30. CAP levels: CON: 0 mg/L; LC: 20 mg/L; and HC: 200 mg/L.

As shown in Figure 2, the LF increased the propionate, decreased acetate percentage and acetate-to-propionate ratio (*p* < 0.05), and tended to decrease valerate percentage (*p* = 0.08). Under dietary factors, LF tended to decrease acetate concentrations in the LC (P tended to decrease valerate percentage (*p* = 0.08). Under dietary factors, LF tended to decrease acetate concenF (*p* < 0.05), and the ammonia concentration in the LF was significantly higher in the HC than in the LF (*p* < 0.05).

### Rumen bacteria

The results of the relative abundance of microbial populations are presented in Table 4. Hyperthermal condition increased the relative abundance of total anaerobic fungi, total methanogens, and *B. fibrisolvens*, while it decreased total protozoa, *F. succinogenes*, and *P. amylophilus* (*p* < 0.05). There is no difference in *R*. *albus*, *R*. *flavefaciens*, and *R. ruminicola* by the effect of hyperthermal condition. The diets significantly decreased total anaerobic fungi, *B. fibrisolvens*, *F. succinogenes*, and *P. amylophilus* (*p* < 0.05) while tended to increase total protozoa and lower *Ruminococcus flavefaciens* (*p* < 0.10). The CAP significantly increased the relative abundance of *B. fibrisolvens* in the LC relative to the CON (*p* < 0.05).

**Table 4:** Microbial relative abundance as affected by incubation temperature, dietary conditions, CAP supplementation, and their interactions.

Item	Temperature (T)			Diet (D)^1^			Capsicum oleoresin (C)^2^			SEM	*p*-Value			Interaction^3^
	39°C	42°C		HF	LF		CON	LC	HC		T	D	C	
Total anaerobic fungi, 10^−5^	0.78	13.97		1.54	0.63		0.83	12.39	11.89	0.17	0.03	0.01	0.38	T × D
Total protozoa, 10^−4^	9.52	0.41		3.64	6.29		3.18	5.97	5.74	1.06	<0.001	0.06	0.17	T × D
Total methanogens, 10^−4^	0.77	3.49		1.91	2.35		1.68	2.07	2.64	0.28	<0.001	0.24	0.12	
*Ruminococcus albus*, 10^−3^	5.01	3.96		6.62	2.42		2.02	2.48	9.07	1.76	0.76	0.26	0.24	
*Ruminococcus flavefaciens*, 10^−3^	9.02	13.44		14.57	7.89		7.91	9.51	16.27	1.83	0.20	0.06	0.12	
*Butyrivibrio fibrisolvens*, 10^−5^	5.25	11.86		9.46	7.65		6.91 ^b^	9.55^a^	9.21^ab^	0.72	0.02	≤0.001	0.03	T × D
*Fibrobacter succinogenes*, 10^−2^	14.54	1.06		10.79	4.81		5.81	7.96	9.63	1.49	<0.001	0.01	0.20	T × D
*Prevotella ruminicola*, 10^−2^	15.52	0.18		11.50	4.21		9.35	9.39	4.82	0.30	<0.001	0.01	0.24	T × D
*Ruminobacter amylophilus*, 10^−2^	1.65	1.26		1.50	1.41		0.75	1.46	2.17	1.96	0.52	0.88	0.17	

1 Dietary conditions (forage/concentrate ratio): LF, 30:70; HF, 70:30.

2 Target CAP concentration (mg/L): CON, 0; LC, 20; HC, 200.

3 Listing only interactions showed a significant effect (*p* ≤ Listing.

a,b Means in the same row with different lowercase superscript letters are significantly different (*p* < 0.05).

## Discussion

The proper achievement of rumen function would depend on temperature and diet composition and affects the ruminants’ health and productivity (Mavrommatis et al., 2021b; Phesatcha et al., 2021; Soltan et al., 2021). CAP has been extensively studied as a feed additive for ruminants *in vivo* (Oh et al., 2015; Castillo-Lopez et al., 2021) and *in vitro* (Busquet et al., 2006); however, little attention has been paid to its effect on rumen fermentation when the rumen is subjected to physiological stress. Therefore, the present study investigated whether CAP could affect rumen function and microbial abundance in the presence and/or absence of hyperthermal condition or under different temperature and dietary conditions. We hypothesized that CAP could maintain rumen fermentation characteristics and microbial abundance.

In this study, temperature and diet significantly affected SCFA concentration in incubation fluid. Although hyperthermal condition reduced SCFA due to a decrease in propionate percentage consistent with the findings of Mahmood et al. (2020)), the temperature did not affect the acetate percentage; it increased the butyrate percentage. Due to milk fat could be synthesized by butyrate (Cheng et al., 2020), there may increase in milk fat under heat stress in dairy cows (Liu et al., 2017). The LF increased SCFA due to increased propionate percentage, similar to the previous study *in vitro* (Natel et al., 2019) and *in vivo* (Ranathunga et al., 2019), and resulted in a difference in acetate-to-propionate ratio. Although CAP could not affect SCFA, it reduced the acetate percentage and tended to increase propionate percentage, consequently reducing acetate-to-propionate ratio (Cardozo et al., 2005). Previously, several studies have shown the exciting phenomenon that CAP cannot affect SCFA and propionate percentage (Busquet et al., 2006; Tager and Krause, 2010; Geron et al., 2019), while only the same effect was produced in LF (Fandiño et al., 2008, 2020). Thus, CAP can only alter SCFA composition under a high-concentrate diet. Also, CAP altered SCFA concentrations in the LC group under hyperthermal condition compared to physiological temperatures. Although CAP decreased acetate-to-propionate ratio and tended to increase the propionate percentage under hyperthermia conditions, it had no effect at physiological temperature. Therefore, it was suggested that CAP altered the fermentation pattern of the rumen under hyperthermia conditions. The effect of CAP on rumen fermentation in different diets was minimal and only trended down the acetate at LF compared to HF. However, the effect of CAP on rumen fermentation *in vivo* under high-concentrate diets may be more due to its stimulation of the digestive tract increasing the secretion of digestive juices, or due to the stimulating nature of capsaicin increasing the duration and frequency of feeding (Patcharatrakul et al., 2020; Castillo-Lopez et al., 2021; Shiiba et al., 2021).

Both diet and temperature increased ammonia concentration; the diet effect was attributed to the crude protein content of the fermentation substrate, while the temperature was responsible for the fermentation inhibition (Castillejos et al., 2005; Phesatcha et al., 2021). Regardless of diet and temperature, the pH of the incubation solution was within the normal range. In this study, CAP could not affect the pH and ammonia concentration of the incubation solution. Although CAP did not affect the increased ammonia concentration due to hyperthermia, ammonia concentration at HF was reduced in the HC, indicating improved microbial ammonia utilization.

Ruminal microorganisms are significant players in ruminal fermentation and are influenced by physicochemical factors, diet changes, and feed additives. Although the literature about the effect of hyperthermia on rumen microbial composition is scarce, it is one of the environmental factors that reduce ruminant performance (Lees et al., 2018; Idris et al., 2021). In the present study, hyperthermal condition increased the relative abundance of anaerobic fungi and methanogenic bacteria. Since anaerobic fungi stimulate the growth of methanogenic bacteria, this may lead to similar results to the previous study (Li et al., 2021). Also, methane production increased due to the action of methanogenic bacteria, although it was not found in this experiment.

On the other hand, hyperthermia reduced the relative abundance of total protozoa consistent with previous studies. It is generally believed that a decrease in the relative abundance of total protozoa leads to a decrease in ammonia concentration based on their phagocytosis of other bacteria (Firkins et al., 2007); however, this has not been found in the present experiment. The latter contradiction may be due to hyperthermia’s inhibition of microbial colonization. Meanwhile, hyperthermia resulted in changes in rumen microbial populations, including a reduction in the relative abundance of *F*. *succinogenes* and *P. ruminicola*, as reported in previous studies (Yadav et al., 2013; Sales et al., 2021). However, in contrast, hyperthermia increased the relative abundance of BF, which may lead to an increase in butyrate concentration.

The microbial community composition due to the diet is more intuitive relative to temperature, mainly regarding the relative abundance of cellulose-degrading bacteria (Kumar et al., 2015). In the present study, HF increased the relative abundance of total anaerobic fungi, *B*. *fibrisolvens*, *F. succinogenes*, and *P. ruminicola*. *Ruminobacter amylophilus* is the primary starch-degrading bacterium in rumen fluid and is known to contribute to propionate production (O’Herrin and Kenealy, 1993), however, HF could not affect the relative abundance of *R. amylolous* but could increase propionate concentration in this experiment. CAP affects various microorganisms, including cellulose-degrading bacteria of rumen origin under culture conditions *in vitro* (Demirtaş, 2020). In this experiment, CAP increased the relative abundance of *B. fibrisolvens*, which was the same as in previous studies *in vivo* (Oh et al., 2015). Additionally, Demirtaş (2020)) reported *B. fibrisolvens* could be stimulated by capsaicin in pure cultures. The role of *B*. *fibrisolvens* is mainly to degrade cellulose and consumes acetate in rumen fermentation for conversion to butyrate (Russell and Strobel, 1989). CAP did not affect butyrate percentage; however, it reduced acetate percentage in this research.

## Conclusion

Hyperthermal condition and dietary factors could alter SCFA concentrations, acetate percentage, propionate percentage, acetate-to-propionate ratio, and ammonia concentrations. The CAP could influence ruminal fermentation *in vitro*, by reducing acetate percentage and acetate-to-propionate ratio. Microbial abundance was mainly influenced by temperature and diet, while the effect of CAP was minimal.

## Data availability statement

The raw data supporting the conclusions of this article will be made available by the authors, without undue reservation.

## Author contributions

ZA and GL wrote the manuscript. MA, UR, and LY revised the manuscript. SG investigated for this research. ZY, TY, and HL provided the methodology for this research. JZ and CC did the formal analysis. All authors have read and agreed to the published version of the manuscript.

## Funding

This work was supported by the earmarked fund for CARS36.

## Conflict of interest

CC was employed by Bright Farming Co. Ltd.

The remaining authors declare that the research was conducted in the absence of any commercial or financial relationships that could be construed as a potential conflict of interest.

## Publisher’s note

All claims expressed in this article are solely those of the authors and do not necessarily represent those of their affiliated organizations, or those of the publisher, the editors and the reviewers. Any product that may be evaluated in this article, or claim that may be made by its manufacturer, is not guaranteed or endorsed by the publisher.
